# Investigation of free cancer cells in peripheral blood using CEA mRNA expression in perioperative colorectal cancer patients

**DOI:** 10.3892/mco.2013.109

**Published:** 2013-04-29

**Authors:** KIICHI NAGAYASU, HIROMITSU KOMIYAMA, SHUN ISHIYAMA, DAI OGURA, RINA TAKAHASHI, YOSHIHIKO TASHIRO, KOICHIRO NIWA, KIICHI SUGIMOTO, YUTAKA KOJIMA, MICHITOSHI GOTO, YUICHI TOMIKI, SHINICHIRO NIWA, KAZUHIRO SAKAMOTO

**Affiliations:** Department of Coloproctological Surgery, Juntendo University, Faculty of Medicine, Bunkyo-ku, Tokyo 113-8431, Japan

**Keywords:** carcinoembryonic antigen mRNA, colorectal cancer, laparoscopic surgery

## Abstract

The aim of this study was to evaluate the impact of laparoscopic surgery (Lap) on circulating free tumor cells in colorectal cancer patients. In this study, we selected carcinoembryonic antigen (CEA) mRNA expression in peripheral blood as the marker of the circulating tumor cells and compared this marker between Lap and open colectomy (OC), to investigate differences due to surgical approach. A total of 50 patients underwent curative surgery for solitary colorectal cancer at our department, between June, 2008 and February, 2011. The patients were divided into OC and Lap groups (25 patients each). Total RNA was extracted subsequent to peripheral blood collection prior to surgery, immediately following surgery and 1, 3 and 7 days after surgery. CEA mRNA was detected with reverse transcription polymerase chain reaction (RT-PCR) and the association between peripheral blood CEA mRNA-positive rate, surgical findings and clinicopathological characteristics was investigated. The peripheral blood CEA mRNA-positive rate was significantly increased immediately after surgery, compared to the preoperative rate (P=0.001), but decreased over time. No significant differences were observed at any blood-sampling time point after postoperative day 1. The positive rate was significantly increased in the OC group immediately after surgery, compared to the preoperative rate (P=0.004). However, there were no significant differences between the rates prior to and immediately after surgery in the Lap group. The patients were then divided into those who were peripheral blood CEA mRNA-positive and -negative after surgery (postoperative positive and negative groups, respectively) and the clinicopathological characteristics were compared. Significant differences were identified between the groups in lower rectal cancer patients and patients with a large intraoperative blood loss (P=0.001 and P=0.01, respectively). In conclusion, in colorectal cancer patients, there were no significant differences in the perioperative peripheral blood CEA mRNA-positive rate or its short-term changes between patients undergoing OC and Lap surgery. It was suggested that Lap is equivalent to OC with regard to free cancer cells.

## Introduction

Approximately 90,000 people develop colorectal cancer and 40,000 succumb to the disease annually in Japan, demonstrating a markedly increased prevalence among Japanese people (1).

Surgical treatment is known to be effective for colorectal cancer and laparoscopic surgery (Lap) became widely used in the first half of the 1990s. Its advantages are the cosmetic appearance of the incision, reduced postoperative pain, early improvement of intestinal movement following surgery and early return to social activities (2). In addition, low-level invasiveness and favorable short-term outcomes have been reported (3–5). Its indications have also been gradually expanded in the Japanese Guidelines for the Treatment of Colorectal Cancer (6). However, reports on the long-term outcomes of advanced colorectal cancer, including transverse colon and rectal cancer, are considered insufficient (7–9).

Free cancer cells have recently attracted attention as a marker indicating micro-metastasis, while the carcinoembryonic antigen (CEA) mRNA has been associated with outcome (10,15–19). It was suggested that the short-term equivalence of Lap and conventional open colectomy (OC) with regard to the oncological viewpoint can be investigated by measuring peripheral blood CEA mRNA expression, which represents free cancer cells, in the perioperative period and comparing it between Lap and OC. For this purpose, we measured peripheral blood CEA mRNA expression in the perioperative period and investigated whether there were differences in the CEA mRNA-positive rate due to the different surgical approaches (OC and Lap), surgical content and clinicopathological characteristics.

## Patients and methods

### 

#### Patients

**Figure 1:**
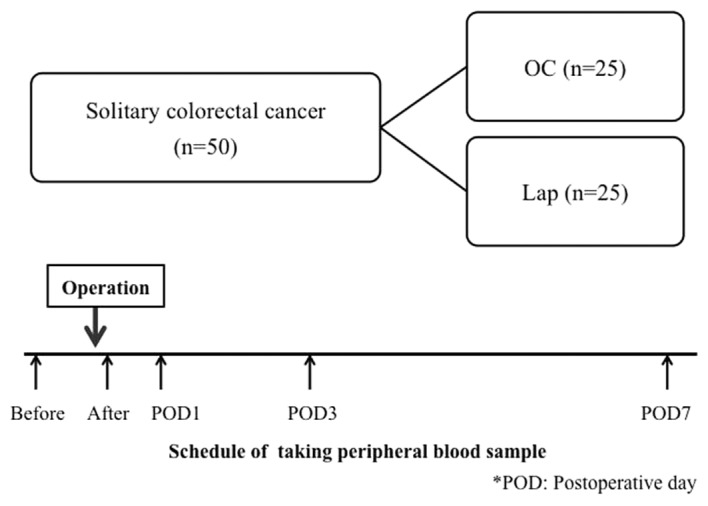
Clinical study design. Fifty patients who underwent curative surgery for solitary colorectal cancer were divided into open colectomy and laparoscopic surgery groups (n=25 each). Peripheral blood was collected from the colorectal cancer patients prior to surgery, immediately after surgery and 1, 3 and 7 days after surgery. Lap, laparoscopic surgery; OC, open colectomy.

A total of 50 patients who underwent curative surgery for solitary colorectal cancer at our department, between June, 2008 and February, 2011 were included in the present study. The patients were divided into OC and Lap groups (25 patients each) (Fig. 1). The exclusion criteria for Lap at our department were as follows: i) large tumor size, exceeding the laparotomy incision (≥8); ii) apparent invasion of other organ(s); iii) stage IV disease, with metastasis to other organ(s); and iv) ileus or intestinal perforation. Accordingly, patients with large tumors (≥8 cm), invasion and/or metastasis to other organ(s) and ileus, for which pressure reduction was impossible, were excluded. Lap was not converted to OC in any of the patients and patients receiving preoperative chemotherapy or radiotherapy were also excluded. Written informed consent was obtained from the patients. The study protocol was approved by the Ethics Committee of Juntendo University (approval no.: 556). Patients who offered their consent selected OC or Lap and were prospectively investigated.

### Methods

#### Blood sampling

Peripheral blood was collected from the colorectal cancer patients prior to surgery, immediately following surgery and 1, 3 and 7 days after surgery (Fig. 1). To prevent contamination with skin tissue during blood sampling, the first 5 ml of blood was discarded. Blood was collected in an RNA stabilizing agent-containing PAXgene Blood RNA tube (Qiagen, Valencia, CA, USA) and stored at −80°C until use.

#### RNA extraction

**Figure 2:**
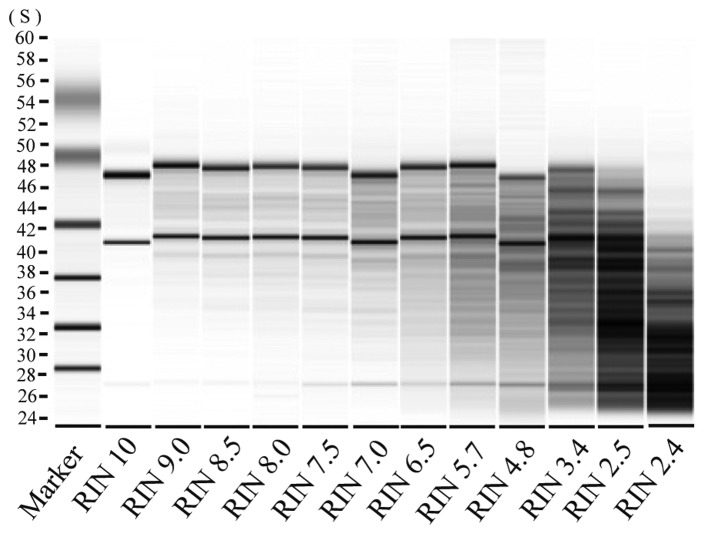
Results of analysis of RNA quality by Agilent bioanalyzer. Bands (28 and 18 sec) become thinner with progressive RNA degradation, the RNA integrity number (RIN) decreases. In this study, in order to preserve analysis accuracy, RNA of RIN ≥6.5 (lane 8) was used for subsequent analysis. S, sec.

Total RNA was extracted from blood samples using the PAXgene Blood RNA kit (Qiagen). To remove contaminating genomic DNA, the blood sample was treated with the RNase-Free DNase I set (Qiagen) and dissolved with 80 *μ*l of RNase-free water. The RNA decomposition level was evaluated using the Bio Analyzer (Agilent, Santa Clara, CA, USA) and samples with an RNA integrity number (RIN) of ≥6.5 were analyzed (Fig. 2).

#### Reverse transcription

Using 100 ng of total RNA as a template, reverse transcription was performed using the SuperScript III First-Strand synthesis system for qRT-PCR (Invitrogen, Carlsbad, CA, USA). Synthesized cDNA was stored at −80°C until use.

#### Real-time PCR

**Figure 3:**
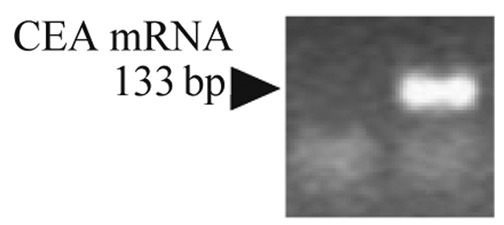
Detection of carcinoembryonic antigen (CEA) in total RNA derived from peripheral blood samples. CEA mRNA in total RNA derived from peripheral blood samples was determined by qRT-PCR with CEA-specific primers. PCR products were subjected to electrophoresis on an agarose gel and stained with ethidium bromide. RNA derived from HT-29 cells was used as a positive amplification control and no template reaction was used as the negative control. CEA was detected in several samples as a single band.

Using 5 *μ*l of cDNA (corresponding to 25 ng of total RNA) as a template, CEA-specific primers and the Power SYBR-Green PCR Master Mix (Applied Biosystems, Foster City, CA, USA) PCR reactions were conducted using the Applied Biosystems 7,500 real-time PCR system (Applied Biosystems) under the reaction condition of 95°C for 10 min, followed by 40 cycles of heat denaturation at 95°C for 15 sec and annealing/elongation at 60°C for 1 min. In order to eliminate false positivity due to non-specific amplification, the experiment was repeated twice and samples confirmed to be amplified in both experiments were accepted as positive (Fig. 3). The CEA-specific primers used are shown below: CEA sense: 5′-GCCTGTTTTGTCTCTAACTTGGC-3′ and antisense: 5′-CAACCAGCACTCCAATCATGAT-3′.

#### Investigation items

Changes in the positive rate over the perioperative period were investigated in the patients. The patients were divided into the OC and Lap groups and patient background factors, including age, gender, tumor site, intraoperative blood loss, operative time, maximum tumor diameter, depth of invasion, lymphatic or vascular invasion, histological type and stage, were investigated. The CEA mRNA-positive rate and its changes over the perioperative period were evaluated.

Patients who were positive and negative for CEA mRNA after surgery were designated as postoperative positive and negative groups, respectively, and the associations with clinicopathological characteristics (age, gender, preoperative CEA level, tumor site, maximum tumor diameter, depth of invasion, histological type, lymphatic or vascular invasion and stage) and surgical factors (surgical approach, intraoperative blood loss, operative time and surgical procedure) were investigated. Staging was performed according to the TNM classification established by the UICC.

#### Statistical analysis

The significance of differences was analyzed by employing the Fisher’s exact, χ^2^, Mann-Whitney U and t-tests, using statistical analysis software SPSS v.17.0 (SPSS Inc., Chicago, IL, USA). P<0.05 was considered to indicate a statistically significant difference.

## Results

**Figure 4:**
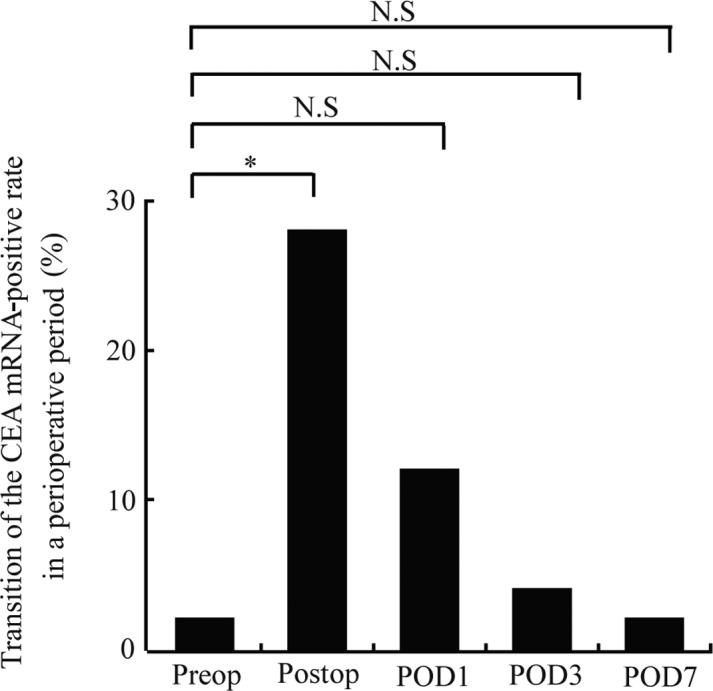
Transition of the carcinoembryonic antigen (CEA) mRNA-positive rate in a perioperative period. The positive rate was significantly increased immediately after surgery (^*^P= 0.001). No significant difference from the preoperative positive rate was noted after postoperative day (POD) 1. N.S., non-significant.

The results from the patients are presented in Table I. Only one patient (2%) was positive for CEA mRNA prior to surgery. The positive rate was significantly increased immediately after surgery to 28% (14 patients) (P=0.001), but decreased over time to 12, 4 and 2% (6, 2 and 1 patient, respectively) at postoperative days 1, 3 and 7, respectively. No significant differences from the preoperative positive rate were noted after postoperative day 1 (Fig. 4).

Differences in the background factors were investigated between the OC and Lap groups. In the OC group, the tumor size was significantly larger compared to that in the Lap group (OC group, 40 mm; Lap group, 28 mm; P=0.04), the intraoperative blood loss was greater (OC group, 200 ml; Lap group, 50 ml; P<0.001) and fewer cases were lymphatic or vascular invasion-positive (P=0.04). However, there were no significant differences between the other factors (Table II).

**Figure 5:**
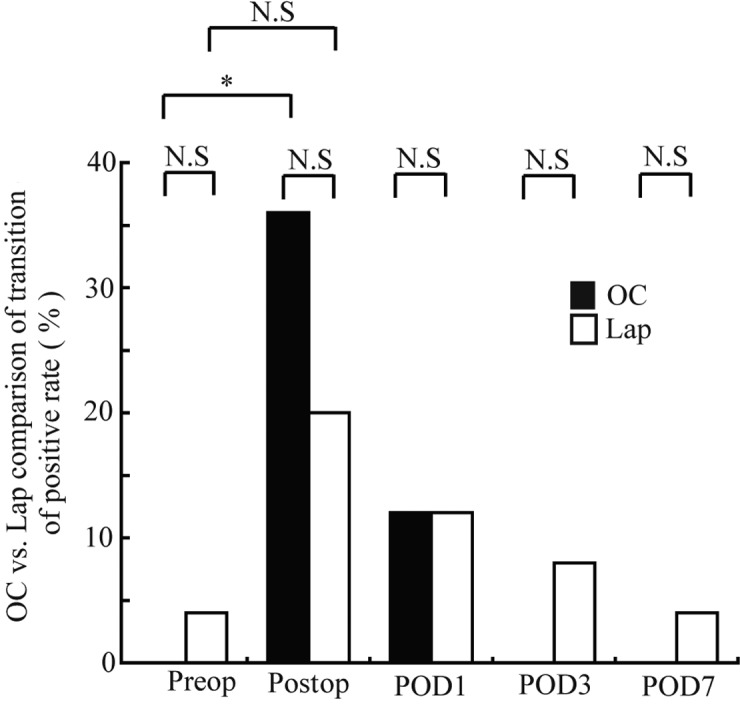
Open colectomy (OC) vs. laparoscopic surgery (Lap) comparison of transition of positive rate. In the OC group, 9 patients (36%) were positive immediately following surgery, showing a significantly higher positive rate compared to the preoperative rate (^*^P=0.004). In the Lap group, the increase in the rate was not significant. No significant difference was noted between the two groups at any blood-sampling time point. N.S., non-significant.

Changes in the peripheral blood CEA mRNA-positive rate over the perioperative period in the OC and Lap groups are shown in Fig. 5. Only one patient (4%) in the Lap group was positive prior to surgery. In the OC group, 9 patients (36%) were positive immediately following surgery, exhibiting a significantly higher positive rate compared to the preoperative rate (P=0.004). In the Lap group, 5 patients (20%) were positive immediately following surgery, although the increase in the rate was not significant. The positive rate decreased over time thereafter in the two groups: 3 patients (12%) in each group were positive at postoperative day 1; 0 (0%) and 2 (8%) at day 3, respectively; and 0 (0%) and 1 (4%) at day 7, respectively, exhibiting no significant differences in the positive rates compared to those prior to surgery. In addition, no significant differences were noted between the 2 groups at any blood-sampling time point.

The patients were divided into those positive and negative for CEA mRNA following surgery, designated as postoperative positive (14 patients) and negative (36 patients) groups, respectively, and the clinicopathological and surgical factors were compared between the groups (Tables III and IV). Regarding tumor site (belonging to the clinicopathological factors), the positive rate was significantly higher in patients with lower rectal cancer compared to those with cancer located elsewhere (P=0.001). Regarding the surgical factors, no significant difference was noted between the surgical approaches (OC vs. Lap), although the intraoperative blood loss was significantly greater in the positive group (P= 0.01) and a significant difference in relation to the surgical procedure was noted (P=0.02). A comparison of the surgical procedures, revealed the positive rate to be significantly higher in the patients treated with inter-sphincteric resection (ISR) and abdominoperineal resection (APR) of the rectum, compared to those treated with other procedures (P=0.01).

## Discussion

Lap for colorectal cancer has become popular, due to its low invasiveness compared to conventional open surgery. However, despite the rapid rise in the popularity of this technique, data on its oncological safety are limited (12,13).

Following the initial success of Smith *et al* (14) in detecting circulating melanoma cells in peripheral blood using RT-PCR, their method has been applied for the detection of cancer cells and cytokeratin 20 and CEA mRNA have been reported to reflect the presence of free cancer cells in peripheral blood. There have been numerous reports on the detection of CEA mRNA using RT-PCR and its association with the outcome (10,15–20). Serum CEA is a tumor-related glycoprotein, most commonly used for the management of colorectal cancer patients in clinical practice. Thus, we used peripheral blood CEA mRNA expression to measure free cancer cells with RT-PCR, as described above. We considered that the short-term equivalence of Lap and OC with regard to the oncological viewpoint may be investigated by measuring peripheral blood CEA mRNA expression, representing free cancer cells, in the perioperative period, during which time the severest surgical stress is experienced.

The association between the timing of detection of free cancer cells in peripheral blood and the outcome varies among different studies. Taniguchi *et al* (16) and Ito *et al* (17) reported that the outcome was significantly poorer in patients who were positive during and immediately after surgery. By contrast, Allen-Mersh *et al* (18) reported a significantly poorer outcome in patients who were positive one day after surgery, and Sadahiro *et al* (19) and Chen *et al* (15) reported that the outcome was significantly poorer in patients who were positive at 7–14 days after surgery. Regarding the time course of positivity, the positive rate was significantly higher immediately following surgery in the OC group (P=0.004), whereas the rate increased, albeit not significantly in the Lap group. The increase in the positive rate may have been due to the surgical operation (tumor dissection) in the two groups, but the rate was lower in the Lap compared to the OC group, suggesting that the non-touch isolation technique was complied with in Lap. The positive rate was decreased at postoperative days 1, 3 and 7 compared to that immediately after surgery in the two groups, while no significant differences were noted in the rate or its time course changes between the groups. It was suggested that there is no difference between the surgical approaches regarding perioperative appearance of free cancer cells.

According to Peach *et al* (10), two processes are responsible for the appearance of free cancer cells. In the first process, cells are released from the primary lesion by a surgical operation, such as dissection and mobilization; in the other process, free cells appear due to disseminating micro-metastasis. Based on these processes, it was hypothesized that the appearance of free cancer cells during and immediately after surgery was significantly affected by the former process, whereas the latter process was responsible for the appearance observed at 3 and 7 days after surgery. However, it must not be ruled out that free cancer cells released by a surgical operation influence micro-metastasis. It has been hypothesized that a surgical stress-induced reduction of immunity contributes to metastasis by free cancer cells entering the circulation during surgery (20) and it has recently been reported that the outcome was significantly poorer in the group in which free cancer cells were detected during or immediately after surgery compared to the group with no detection of free cancer cells (10).

We considered that postoperative positivity is important, rather than the timing of blood sampling. We compared patients who were positive at least once in the period immediately following surgery and thereafter (postoperative positive group) with those who showed no positivity (postoperative negative group). As demonstrated by the results, significant differences were noted in patients in the postoperative positive group in whom the cancer was located in the lower rectum (P=0.0001), with a large volume of intraoperative blood loss (P=0.001) and in whom the surgical procedure was ISR or APR (P=0.01), although no significant difference was observed between the surgical approaches (P=0.13). Regarding the tumor site, a significant difference was noted in patients with lower rectal cancer, but not in those with cancer located elsewhere. Similarly, a significant difference was noted in patients treated by ISR or APR, but not in those treated by other surgical procedures. It was assumed that cancer cells are readily released from the lower rectum due to the absence of serosa, which is a patient factor. In addition, this procedure is more complex and the dissection distance is longer in surgery for lower rectal cancer compared to other colorectal cancers, which are surgical factors. ISR or APR is frequently employed for lower rectal cancer. The blood loss is generally greater with this procedure compared to other procedures. Although blood loss is dependent on the location of the tumor (lower rectal cancer), it may serve as an index from the oncological viewpoint. No significant differences were noted in the background clinicopathological characteristics of the patients between the Lap and OC groups and the blood loss was significantly lower in the Lap compared to the OC group. It was suggested that Lap for colorectal cancer is better or at least equivalent to OC with regard to peripheral blood free cancer cells, which is considered as micro-metastasis.

In conclusion, in colorectal cancer patients, there were no significant differences in the perioperative peripheral blood CEA mRNA-positive rate, or its short-term changes, between the patients receiving open and laparoscopic surgeries. It was suggested that Lap is equivalent to OC with regard to free cancer cells. Additional studies are necessary in order to assess more cases, verify the findings and investigate their association with long-term outcome.

## Figures and Tables

**Table I:** Comparative results from open colectomy and laparoscopic surgery groups on different postoperative days (POD).

No.	Before	After	POD1	POD3	POD7
Open colectomy group					
1	−	−	−	−	−
2	−	+	+	−	−
3	−	+	+	−	−
4	−	−	−	−	−
5	−	−	−	−	−
6	−	−	−	−	−
7	−	−	−	−	−
8	−	−	−	−	−
9	−	−	−	−	−
10	−	+	−	−	−
11	−	+	−	−	−
12	−	−	−	−	−
13	−	−	−	−	−
14	−	+	+	−	−
15	−	+	−	−	−
16	−	−	−	−	−
17	−	−	−	−	−
18	−	+	−	−	−
19	−	+	−	−	−
20	−	−	−	−	−
21	−	+	−	−	−
22	−	−	−	−	−
23	−	−	−	−	−
24	−	−	−	−	−
25	−	−	−	−	−
Total	0/25 (0%)	9/25 (36%)	3/25 (12%)	0/25 (0%)	0/25 (0%)
Laparoscopic surgery group					
1	−	−	−	−	−
2	−	−	−	−	−
3	−	−	−	−	−
4	−	−	−	−	−
5	−	−	−	−	−
6	−	+	−	−	−
7	−	+	+	−	−
8	−	−	−	−	−
9	−	−	−	−	−
10	−	−	−	−	−
11	−	+	+	−	−
12	−	+	−	−	−
13	−	−	−	−	−
14	+	−	−	+	−
15	−	−	−	−	−
16	−	−	−	−	−
17	−	−	−	−	−
18	−	−	−	−	−
19	−	−	−	−	−
20	−	−	+	+	+
21	−	−	−	−	−
22	−	−	−	−	−
23	−	−	−	−	−
24	−	−	−	−	−
25	−	+	−	−	−
Total	1/25 (4%)	5/25 (25%)	3/25 (12%)	2/25 (8%)	1/25 (4%)

**Table II:** OC vs. Lap patient characteristics.

Variables	OC (n=25)	Lap (n=25)	P-value
Age (years)	66.7±11.0	64.0±11.3	0.41
Gender			
Male/female	11/14	17/8	0.15
Tumor site			
Right colon/other	6/19	9/16	0.27
Left colon/other	9/16	7/18	0.38
Upper rectum/other	5/20	7/18	0.37
Lower rectum/other	5/20	2/23	0.21
Blood loss in ml (range)	200 (10–880)	50 (15–255)	<0.00
Time of operation in min (average)	140–584 (235)	210–510 (263)	0.07
Tumor size in mm (average)	11–87 (40)	10–85 (28)	0.04
Depth of invasion			
T1-2/T3-4	9/16	15/10	0.16
Lymphatic or venous invasion			
Absent/present	12/13	5/20	0.04
Histological type			
TUB1/TUB2-POR	8/17	11/14	0.28

OC, open colectomy; Lap, laparoscopic surgery; TUB, tubular adenocarcinoma; POR, poorly differentiated adenocarcinoma.

**Table III:** Positive vs. negative group clinicopathological characteristics.

Variables	Positive group 1^a^ (n=14)	Negative group 2^b^ (n=36)	P-value
Age (years)	62.9±10.5	66.3±11.3	0.34
Gender			
Male/female	7/7	15/21	0.75
Preoperative serum CEA in ng/ml (average)	0.3–68.5 (4.4)	0.7–14.3 (2.9)	0.20
Tumor site			
Right colon/other	4/10	11/25	0.59
Left colon/other	3/11	13/23	0.26
Upper rectum/other	1/13	11/25	0.08
Lower rectum/other	6/8	1/35	0.001
Tumor size in mm (average)	20–45 (32)	10–87 (34)	0.93
Depth of invasion			
T0-2/T3-4	7/7	17/19	0.55
Histological type			
TUB1/TUB2-POR	5/9	14/22	0.55
Lymphatic or venous invasion			
Absent/present	5/9	12/24	0.56

a Positive group 1, cases positive at least once after the operation;

b Negative group 2, cases negative after the operation. CEA, carcinoembryonic antigen; TUB, tubular adenocarcinoma; POR, poorly differentiated adenocarcinoma.

**Table IV:** Positive vs. negative group surgical factors.

Variables	Positive group (n=14)	Negative group (n=36)	P-value
Approach			
OC/Lap	9/5	15/21	0.13
Blood loss in ml (average)	30–880 (182)	10–500 (80)	0.01
Time of operation in min (average)	190–430 (261)	140–584 (250)	0.15
Procedure			
Partial resection	6	19	
Hemicolectomy	1	3	
Anterior resection^a^	2	13	0.02
ISR or APR^b^	5	2	
ISR or APR/other	5/9	2/34	0.01

a Including low anterior resection.

b Intersphincteric or abdominoperineal resection. OC, open colectomy; Lap, laparoscopic surgery; ISR, intersphincteric resection; APR, abdominoperineal resection.

## Abbreviations:

Lap: laparoscopic surgery;
OC: open colectomy;
RIN: RNA integrity number;
RT-PCR: reverse transcription-polymerase chain reaction;
ISR: intersphincteric resection;
APR: abdominoperitoneal resection
